# Multiplex quantitative analysis of stroma-mediated cancer cell invasion, matrix remodeling, and drug response in a 3D co-culture model of pancreatic tumor spheroids and stellate cells

**DOI:** 10.1186/s13046-019-1225-9

**Published:** 2019-06-14

**Authors:** Hyun Ju Hwang, Min-Suk Oh, Dong Woo Lee, Hyo-Jeong Kuh

**Affiliations:** 1 Department of Biomedicine & Health Sciences, Graduate School, The Catholic University of Korea, Seoul, Republic of Korea; 2 Departments of Biomedical Engineering, Konyang University, Daejeon, Republic of Korea; 3 Medical & Bio Device, #B-9, 145 Gwanggyo-ro, Suwon, Republic of Korea; 4 Cancer Evolution Research Center, College of Medicine, The Catholic University of Korea, Seoul, Republic of Korea; 5 Department of Medical Life Sciences, College of Medicine, The Catholic University of Korea, 222 Banpo-daero, Seocho-ku, Seoul, 06591 Republic of Korea

**Keywords:** Tumor spheroids, Pancreatic stellate cell, Tumor microenvironment, Matrix remodeling, 3D co-culture, Cancer invasion, Paclitaxel

## Abstract

**Background:**

Pancreatic ductal adenocarcinoma (PDAC) is a stroma-rich carcinoma, and pancreatic stellate cells (PSCs) are a major component of this dense stroma. PSCs play significant roles in metastatic progression and chemoresistance through cross-talk with cancer cells. Preclinical in vitro tumor model of invasive phenotype should incorporate three-dimensional (3D) culture of cancer cells and PSCs in extracellular matrix (ECM) for clinical relevance and predictability.

**Methods:**

PANC-1 cells were cultured as tumor spheroids (TSs) using our previously developed minipillar chips, and co-cultured with PSCs, both embedded in collagen gels. Effects of PSC co-culture on ECM fiber network, invasive migration of cancer cells, and expression of epithelial-mesenchymal transition (EMT)-related proteins were examined. Conditioned media was also analyzed for secreted factors involved in cancer cell-PSC interactions. Inhibitory effect on cancer cell invasion was compared between gemcitabine and paclitaxel at an equitoxic concentration in PANC-1 TSs co-cultured with PSCs.

**Results:**

Co-culture condition was optimized for the growth of TSs, activation of PSCs, and their interaction. Increase in cancer cell invasion via ECM remodeling, invadopodia formation and EMT, as well as drug resistance was recapitulated in the TS-PSC co-culture, and appeared to be mediated by cancer cell-PSC interaction via multiple secreted factors, including IL-6, IL-8, IGF-1, EGF, TIMP-1, uPA, PAI-1, and TSP-1. Compared to gemcitabine, paclitaxel showed a greater anti-invasive activity, which was attributed to suppresion of invadopodia formation in cancer cells as well as to PSC-specific cytotoxicity abrogating its paracrine signaling.

**Conclusions:**

Here, we established 3D co-culture of TSs of PANC-1 cells and PSCs using minipillar histochips as a novel tumoroid model of PDAC. Our results indicate usefulness of the present co-culture model and multiplex quantitative analysis method not only in studying the role of PSCs and their interactions with tumor cell towards metastatic progression, but also in the drug evaluation of stroma-targeting drugs.

**Electronic supplementary material:**

The online version of this article (10.1186/s13046-019-1225-9) contains supplementary material, which is available to authorized users.

## Background

Pancreatic ductal adenocarcinoma (PDAC) is an aggressive malignant tumor of the exocrine pancreas with a 5-year survival rate of less than 5% [1]. Only 15–20% of the patients are candidates for curative surgical resection [2]. In patients with unresectable or metastatic disease, gemcitabine (GEM) has been the cornerstone of treatment, despite only a small advantage in terms of survival [3].

PDAC is one of the most stroma-rich carcinomas, and pancreatic stellate cells (PSCs) are a major component of this dense stroma [4]. PSCs differentiate into cancer-associated fibroblasts (CAFs) and have a strong impact on tumor progression through cross-talk with cancer cells [5]. PSC-derived secretory factors including growth factors are released into the tumor microenvironment (TME) and promote epithelial-mesenchymal transition (EMT), which in turn induces cancer invasion and drug resistance [6]. PSCs also participate in remodeling of the ECM [7] and exert a physical influence stimulating cell migration [8]. These characteristics of PSCs highlight the importance of PSCs in tumor model for human PDAC.

In vitro tumor models may be useful for studying the effects of PSCs on tumor growth, invasion, and drug resistance. Traditional two-dimensional (2D) cultures do not take into account the in vivo conditions of the TME [9] and yield data with minimal clinical relevance. On the other hand, three-dimensional (3D) tumor models such as tumor spheroids (TSs) retain important 3D characteristics of in vivo tumors and TME and recapitulate the molecular signature and behavior of cancer cells [10–12]. In particular, spheroid cultures embedded in suitable matrix such as collagen allow 3D cell-matrix interactions, hence, they are increasingly used to study cell-ECM interactions [13], local invasion [14], migration [15], and drug resistance [16].

We were to develop a 3D co-culture model for high-content analysis of cellular processes such as cell invasion, matrix remodeling and drug response, resulting from the interaction between cancer cells and PSCs. We used our previously developed minipillar chip designed for the culture of matrix-embedded TSs [17, 18] and culture conditions were further optimized for 3D reciprocal interactions between TSs of PANC-1 pancreatic cancer cells and PSCs. Under these conditions, TSs acquired a more invasive phenotype with a progressive EMT signature, and promoted the remodeling of the ECM. Paclitaxel (PTX), compared to GEM, had a superior inhibitory effect on the EMT signature and invasiveness of cancer cells. Overall, our minipillar co-culture model represents a novel method for evaluation of stroma-mediated cancer cell invasion, matrix remodeling and differential drug sensitivity in pancreatic tumor.

## Methods

### Cell culture and reagents

Human pancreatic cancer cell lines, MIAPaCa-2, AsPC-1, PANC-1, Capan-1 and BxPC-3 were purchased from American Type Culture Collection (ATCC, USA). PSC, human pancreatic stellate cell line, was obtained from ScienCell (HPaSteC, #3830, Carlsbad, CA). MIAPaCa-2 and PANC-1 cells and PSCs were maintained in high glucose DMEM (Hyclone, Logan, UT) and AsPC-1, BxPC-3 and Capan-1 cells in RPMI-1640 medium (Gibco, Grand Island, NY); both media were supplemented with 100 μg/mL streptomycin, 100 units/mL penicillin, 250 ng/mL amphotericin B and 10% fetal bovine serum (FBS, Welgene, Daegu, Korea). Cells cultures were maintained at 37 °C humidified atmosphere (5% CO_2_ /95% air).

### Preparation of minipillar array chips

Minipillar array chip was custom-made (MBD Co., Suwon, Korea). Detailed specification and preparation of the minipillar array chip has been reported [18, 19]. Briefly, the minipillar array chip consists of a pair of reusable base chips (top and bottom chip) and disposable minipillars (Additional file 1: Figure S1-a, b). Twenty-five minipillars were arranged in 9 mm-pitch for cell culturing in 96-well plates and in 3.2 mm-pitch for preparing cryo-sections (Additional file 1: Figure S1-c). The minipillars were sterilized by boiling in 70% ethanol for 30 min followed by ultraviolet irradiation (Ultraviolet Crosslinkers, UVP®, CX-2000). Base chips were autoclaved for sterilization.

### Culture and drug expose condition for co-culture of TSs and PSCs

Cancer cells and PSCs were suspended at 8 × 10^5^ cells/mL and 4 × 10^4^ cells/mL, respectively, in 2.33 mg/ml collagen I solution (Rat tail tendon type I collagen, BD Biosciences, San Jose, CA) [20]. Cancer cells were loaded on the tips of minipillars at 1.6 × 10^3^ cells/2 μL and PSCs into each well of 96-well plates in 1.6 × 10^3^ cells/40 μL, then, both were allowed to gel before adding culture media. Co-culture was done by transferring the pillar chip containing cancer cells to 96-well plates containing PSCs. Cancer cells grew into spheroids in the collagen matrix and medium change was done every 2 days. Drug treatment was done by exposing the cells after 6-days of culture in the drug-containing media for 72 h in 96 well plates. Cell viability was determined using acid phosphatase (APH) assay and calcein AM/PIstaining (BDA-1000, BIOMAX, Seoul, Korea), according to the procedure provided by the manufacturer.

### Preparation of the frozen sections of TSs

The Frozen sections of TSs grown on minipillar chips were prepared as previously described. Briefly, TSs was frozen on minipillar chips in the vapor of liquid nitrogen. To prepare frozen blocks of TSs, minipillars were rearranged from 9 mm-pitch to 3.2 mm-pitch array (Additional file 1: Figure S1-c) and embedded in OCT compound using embedding jig assembly (EM001, MBD Co.) and freezing at − 20 °C for 20 min in a cryotome chamber. After 20 min, the pillar array chip was carefully removed, leaving the TSs collagen cap array, in the frozen OCT block. The frozen TS blocks were cut into 5 μm-thick sections.

### Immunohistochemical staining and analysis

Immunostaining in TSs and PSCs was done either in 96-well plates during cultivation or on cryo-sections using antibody or fluorescent dye. For in-well staining, TSs were first fixed in 3.7% formaldehyde for 20 mins and further incubated with 0.5% Triton X-100 for another 30 mins. Exposure to primary antibodies against alpha-smooth muscle actin (α-SMA) (1:100, Abcam, ab5694), type I collagen (1:300, Abcam, ab34710), transforming growth factor beta-1 (TGF-β1) (1:50, Abcam, ab92486), membrane-type 1 matrix metalloproteinase (MT1-MMP) (1:100, Abcam, ab78738), vimentin (1:500, Abcam, ab92547) and wingless-related integration site-2 (Wnt2) (1:100, Abcam, ab27794) was done at 4 °C in a humidified chamber for 2 days. After blocking non-specific binding using 10% normal goat serum for 2 h, visualization was done using secondary antibodies conjugated with either Alexa Fluor 594 (1:1000, Thermo Scientific, A27016) or 488 (1:1000, Thermo Scientific, A11034). F-actin was stained with rhodamine phalloidin (1:1000, Invitrogen, R415). Samples were counter-stained with DAPI (1:2000, Sigma-Aldrich, D9564) and subjected for confocal microscopy (LSM 800 W/Airyscan, Carl Zeiss, Oberkochen, Germany).

Immunostaining on cryo-sections of TSs was preceded by collagen removal by 20 min-incubation in cold-DPBS followed by fixation in 95% ethanol for 15 min. Incubation with primary antibodies against α-SMA (1:100, Abcam), β-catenin (1:100, Cell Signaling, 8480), epithelial-cadherin (E-cadherin) (1:500, Cell Signaling, #3195), fibronectin (1:600, Abcam, ab2413), Ki-67 (1:400, Santa Cruz, sc-15,402), TGF-β1 (1:200, Abcam), type I collagen (1:600, Abcam) and vimentin (1:600, Abcam) was done at 4 °C in a humidified chamber overnight. After blocking non-specific binding using 10% normal goat serum for 60 min, visualization was done using secondary antibody conjugated with Alexa Fluor 594 (1:2000, Thermo Scientific). Sections were then counter-stained with DAPI and mounted for confocal microscopy (Carl Zeiss).

### Image analysis

The fluorescence intensity was determined using ZEN software (Carl Zeiss). Morphological analysis and object counting was done by using ImageJ (NIH) software. The apparent diameter (D) of the TSs was calculated from the area measured using ImageJ and the equation, D = 2 × (area/π)^1/2^ assuming spherical shapes of TSs. Cell aggregates with a diameter greater than 40 μm were considered as full spheroids. Surface roughness of the TSs was expressed using circularity defined as, 4π × area/perimeter^2^. The morphological changes of spheroids and nuclei in cells invading into surrounding matrix were analyzed by using the aspect ratio defined as ratio of major vs minor axis. Structural organization of matrix fibers was analyzed for thickness and orientation. The orientation angle of collagen fibers was set at zero to the growing axis of invadopodia. The degree of cell invasion was determined by the number of dispersed single cells in the matrix outside spheroids. The regions of interest (ROI) for image analysis were selected randomly as the area containing 30–50 spheroids on the designated sections, unless otherwise indicated.

### Western blot analysis

The cultured TSs were incubated in 0.1% collagenase D (Sigma-Aldrich, 11,088,858,001) at 37 °C for 30 min and lysed using a RIPA buffer. Total protein in lysates was quantified by BCA assay (Pierce™ BCA Protein Assay Kit, Thermo Scientific, #23227), 25 μg of total protein were resolved on 6 and 10% SDS-PAGE gel under reducing conditions, and transferred onto PVDF membranes (Immun-Blot® PVDF Membrane, BIO-RAD, #1620176). The membranes were blocked with 5% skim milk in TBS with 0.05% Tween-20 (TBS-T) at room temperature, and incubated with antibodies against α-SMA (1:1000, Abcam), β-catenin (1:500, Cell Signaling), E-cadherin (1:500, Cell Signaling), TGF-β1 (1:1000, Abcam), and vimentin (1:5000, Abcam) and β-actin (1:5000, Thermo Scientific, MA5–13739) at 4 °C overnight. After exposing to horseradish peroxidase (HRP)-conjugated secondary antibody, proteins were visualized by chemiluminescent substrate (SuperSignal™ West Pico PLUS, Thermo Scientific, #34580). The band intensity was analyzed using GeneTools software (Syngene, Cambridge, UK) and expression level was expressed as the ratio of target protein relative to β-actin. The value of the mono-culture group was set to 1 and the expression ratios of the co-culture group were compared.

### Human secretome array analysis

The expression levels of chemokines and cytokines were analyzed using a Human Cytokine Antibody Array (C5) and a customized antibody array that includes ten factors known for their roles in EMT (tissue inhibitors of metalloproteinases-1, TIMP-1; urokinase plasminogen activator, uPA; plasminogen activator inhibitor-1, PAI-1; thrombospondon-1, TSP-1; MMP-2; MMP-9; E-cadherin; neural-cadherin, N-cadherin; Latent TGF-β1; stromal cell derived factor-1, SDF-1), (RayBiotech, GA). According to the manufacturer’s instruction, antibody-embedded membranes were incubated with 1 ml of conditioned media (CM) at 4 °C overnights, followed by incubation with biotin-conjugated detection antibody cocktail and diluted HRP-streptavidin at room temperature. Proteins were then visualized using a chemiluminescent substrate reagent. The signal intensities were quantified using GeneTools software.

## Results

### Optimization of the 3D co-culture conditions for TSs and PSCs

**Fig. 1:**
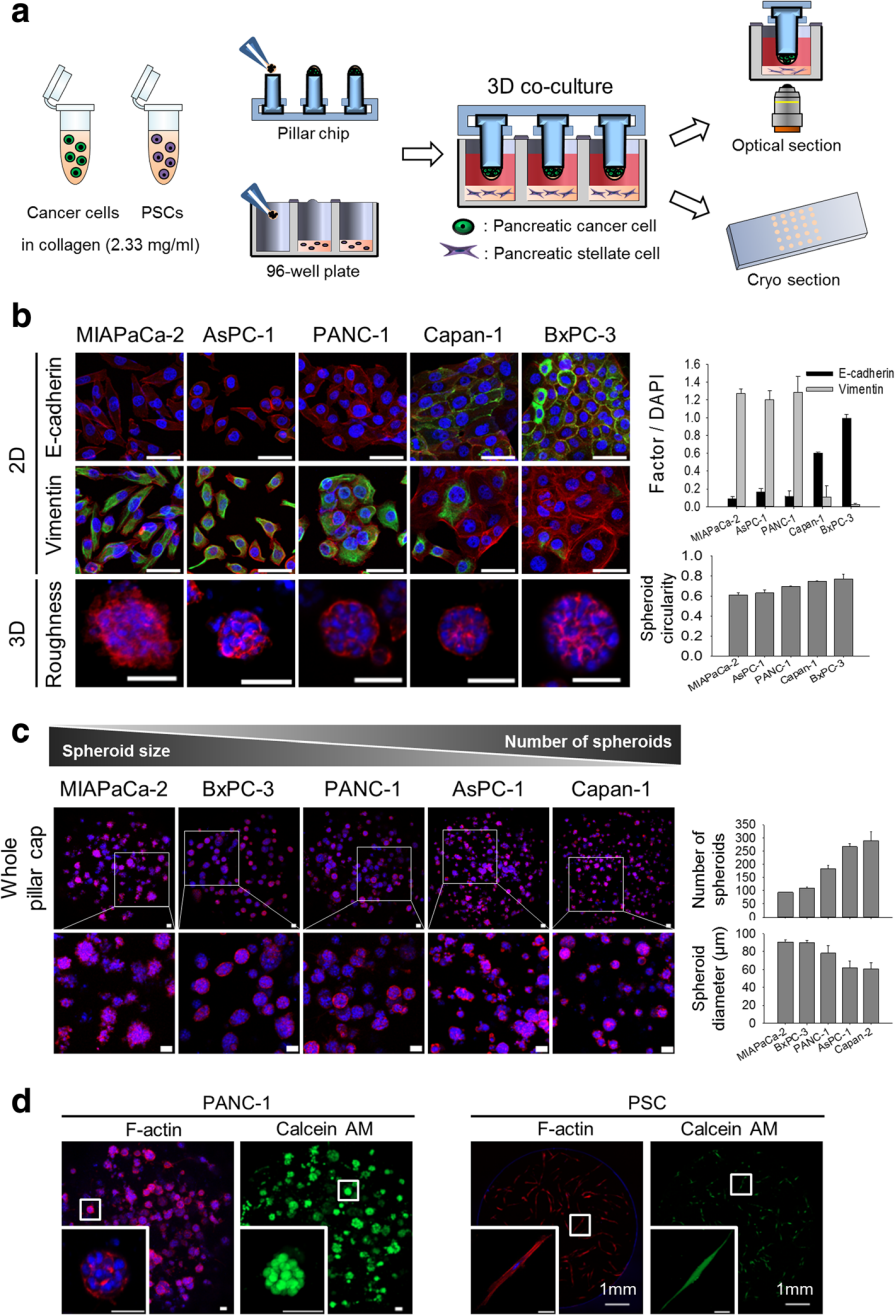
Optimization of co-culture conditions for TSs and PSCs. **a** A schematic illustration of cancer cells-PSCs co-culture using minipillar chips and 96-well plates and subsequent confocal microscopy following immunofluorescence staining. **b** The relationship between EMT status (levels of E-cadherin and vimentin expression; green) and spheroid surface roughness among five pancreatic cancer cell lines. **c** Comparison of the number and size of spheroids among five pancreatic cancer cell lines. **d** Morphology and viability (calcein AM) of TSs and PSCs when co-cultured. Cells were grown in the absence (**b** and **c**) and presence (**d**) of PSCs for 6 days. Staining of whole TSs and PSCs was carried out during cultivation in the well plates, and optical sections were acquired at 2 μm (**b**), 10 μm (**c**) and 5 μm (**d**) intervals and stacked into a z-projection. Data represent the mean ± SD of three independent experiments. Scale bars: 50 μm

We established 3D co-culture of cancer cells and PSCs using a minipillar array chip combined with 96-well plates, in which reciprocal paracrine interaction were optimized (Fig. 1-a). Five pancreatic cancer cell lines with differential mesenchymal status were used to culture 3D spheroids in collagen matrix and the surface roughness of TSs was compared by using circularity (Fig. 1-b). Spheroid circularity correlated with the mesenchymal status of cell lines, i.e., MIAPaCa-2, AsPC-1, and PANC-1 cells with strong expression of vimentin formed spheroids with rather rough-surface, compared to Capan-1 and BxPC-3 cells with strong expression of E-cadherin. Growth characteristics of TSs also varied among the cell lines showing a range of the number and size of spheroids formed after 6 days of culture (Fig. 1-c). MIAPaCa-2 and BxPC-3 cells formed a smaller number of spheroids being larger in size, whereas AsPC-1 and Capan-1 cells formed a greater number of spheroids being smaller in size. PANC-1 spheroids were ranked somewhere in the middle among the five cell lines in both the number and size of spheroids as well as surface roughness of the spheroids. We selected PANC-1 cells as a model cell line for further experiments. Under co-culture conditions, PANC-1 TSs and PSCs were viable and evenly distributed in the embedding collagen as evidenced by cell morphology and calcein AM staining after 6 days of culture (Fig. 1-d).

### Activation of PSCs during co-culture with PANC-1 TSs

**Fig. 2:**
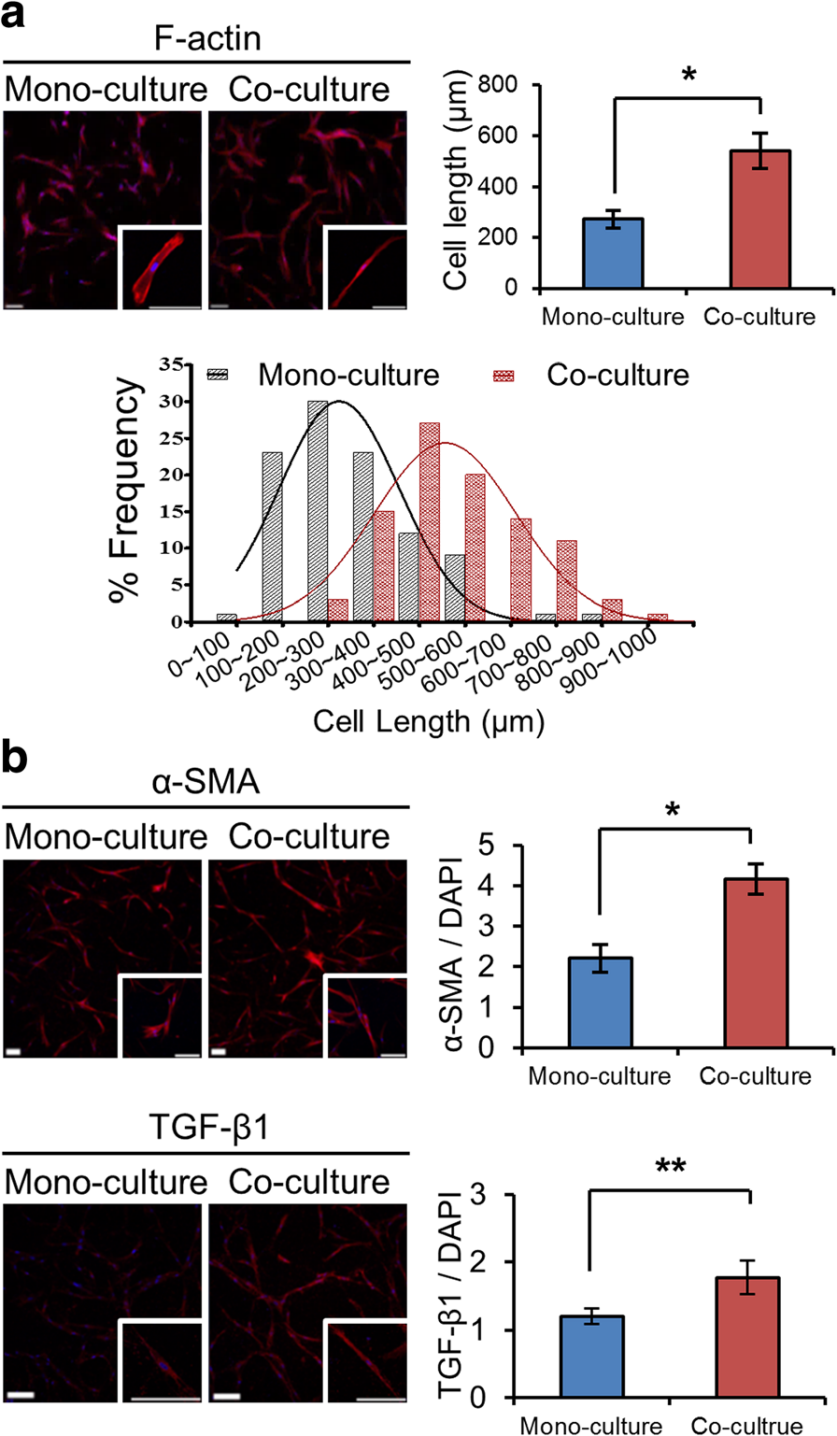
Activation of PSCs when co-cultured with PANC-1 spheroids. **a** Changes in the cell shape and length when co-cultured with PANC-1 spheroids. **b** The expression of α-SMA and TGF-β1 increased in PSCs co-cultured with TSs. Immunostaining was done after 7 day of culture in 96-well plates. Optical sections were acquired at 5 μm (**a**) and 1.5 μm (**b**) intervals and stacked into a z-projection. Data represent the mean ± SD of three independent experiments. Minimum of 50 cells were analyzed per field. Scale bars: 200 μm. * *p* < 0.05, **p < 0.01

PSCs were cultured collagen-embedded in 96-well plates, and their growth and morphology were monitored for 7 days. When co-cultured with PANC-1 TSs, PSCs showed a significant morphological change — to an elongated spindle shape (mean length, 314 vs. 631 μm; Fig. 2-a) — and increased expression of α-SMA and TGF-β1 by 1.88- and 1.47-fold, respectively (Fig. 2-b). These results indicated that PSCs were differentiated to CAFs and activated to a myofibroblastic state through the interaction with pancreatic cancer cells in our co-culture system.

### ECM remodeling around PANC-1 TSs by PSC co-culture

**Fig. 3:**
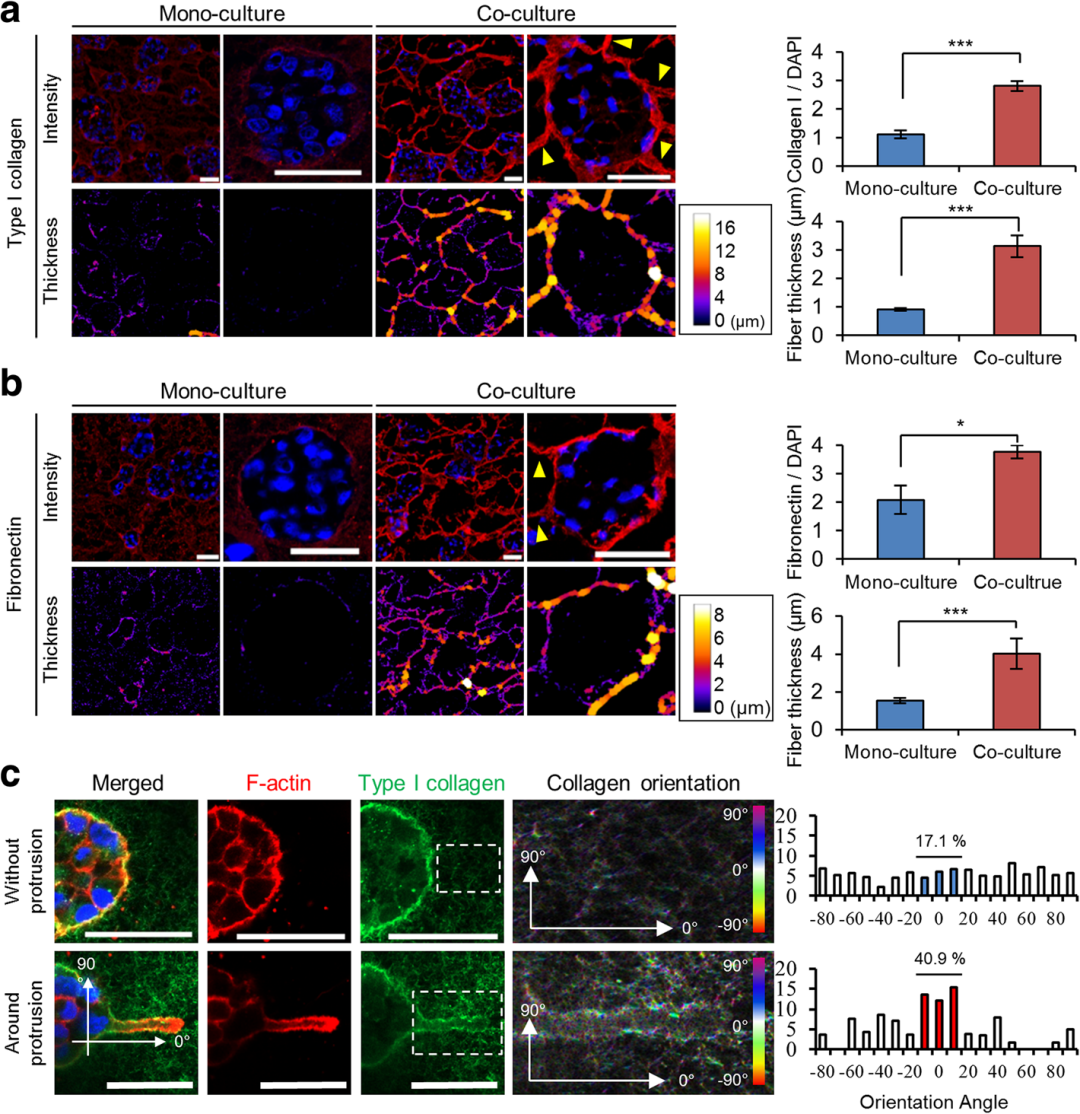
Changes in distribution and morphology of matrix protein fibers. The effects of PSC co-culture on the expression level and thickness of type I collagen (**a**) and fibronectin (**b**). Yellow arrowheads indicate the ECM bundle found in the periphery of TSs. **c** Difference angle distribution of collagen fibers between normal (non-invaded) matrix and matrix proximal to membrane protrusion (Invadopodia). After 7 days of culture, immunostaining was performed on cryo-sections of TSs (**a** and **b**) or on the whole TSs (**c**). Optical sections were acquired at 1 μm intervals and stacked into a z-projection. Data represent the mean ± SD of three independent experiments. Scale bars: 50 μm. * p < 0.05, ***p < 0.001

PANC-1 cells secreted and deposited matrix proteins type I collagen and fibronectin within spheroids as well as in the extracellular space surrounding them (Fig. 3-a, b). During the co-culture of TSs with PSCs, type I collagen deposition was significantly elevated particularly at the spheroid boundary, thus resulting in greater fiber thickness (Fig. 3-a). Similar changes were observed in fibronectin expression (Fig. 3-b). Analysis of collagen fiber orientation surrounding actin-rich protrusion of cell membrane revealed specific changes in the angle distribution which was paralleled the growing axis (Fig. 3-c), suggesting that collagen matrix remodeling took place simultaneously with membrane protrusions and elongation. Out-growing tracks of bundled collagen presented in Fig. 3-a (yellow arrowheads) corresponded to the matrix remodeling in the direction of growing protrusion (Fig. 3-c). These actin-rich membrane protrusions showed the expression of vimentin intermediate filament and MT1-MMP (Additional file 2: Figure S2), hence were defined as invadopodia [21, 22].

### The effect of PSC co-culture on growth and invasion of PANC-1 TSs

**Fig. 4:**
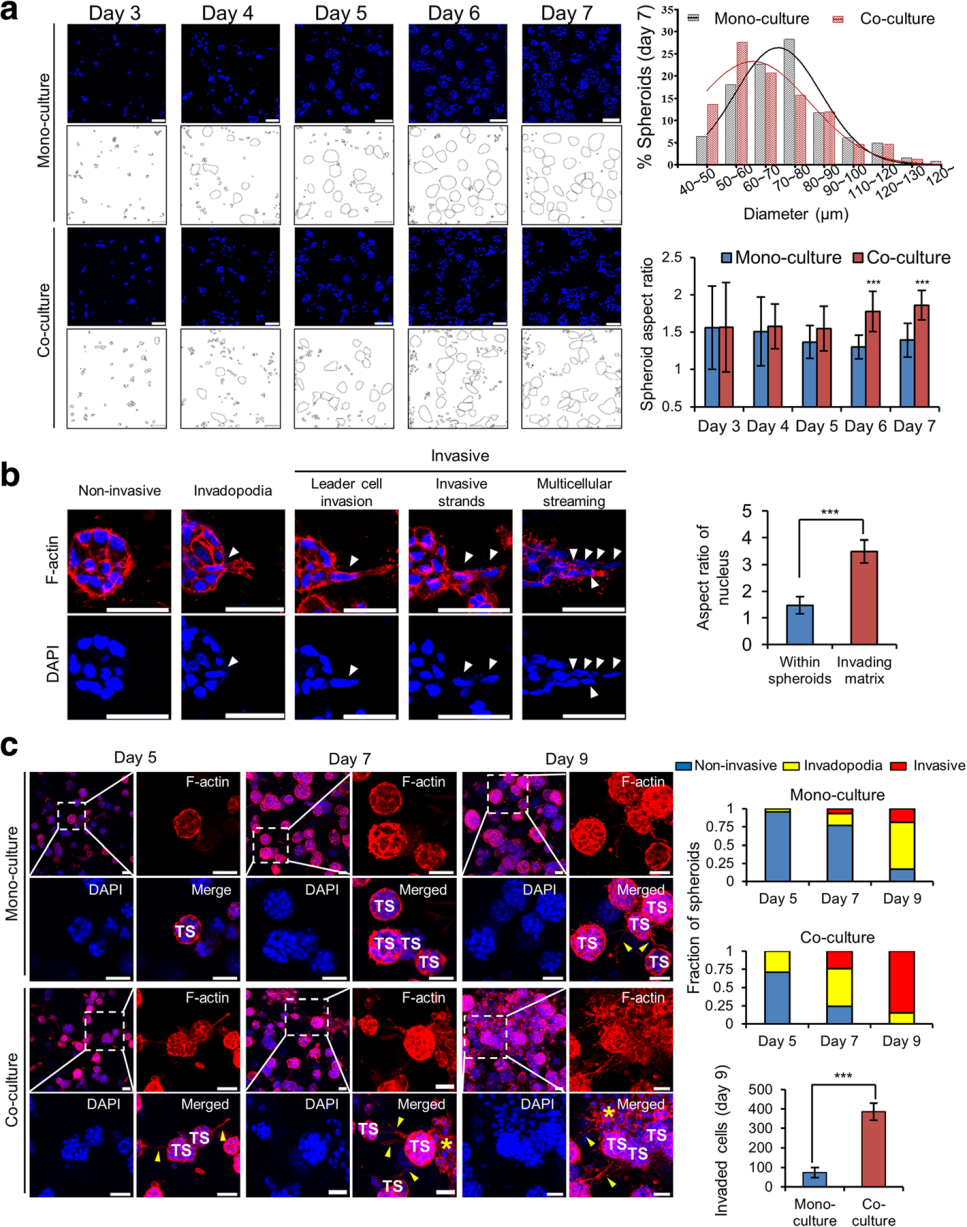
The effect of PSC co-culture on cell invasion from PANC-1 TSs. **a** Changes in size and shape of the spheroids under PSC co-culture conditions. **b** Three distinct stages of progressive invasion of cancer cells out of TSs. The cells with nuclear elongation are indicated by white arrowheads and nuclear aspect ratio was compared between cells remaining within spheroids and cells invading matrix. **c** Increased fraction of TSs with invasive morphology under PSC co-culture conditions. Invadopodia (yellow arrowheads) and dispersed single cells (yellow asterisks) can be seen around some spheroids. Sectional areas containing minimum of 30 spheroids were analyzed for their invasion stage of non-invasive, invadopodia, or invasive as defined in (**b**). The cell invasion was compared calculated the number of dispersed single cells found in the matrix outside spheroids and compared between mono-culture TSs and TSs co-cultured with PSCs on day 9 of culture. Nuclear DAPI (blue) or F-actin (red) staining was performed on cryo-sections of TS (**a**) or on whole TSs (**b** and **c**). Confocal optical sections were acquired at 1 μm (a and b) or 7 μm (**c**) intervals and stacked into a z-projection. TS: tumor spheroid. Data represent the mean ± SD of three independent experiments. Scale bars: 50 μm. ***p < 0.001

PANC-1 cells, when embedded in the collagen gel, manifested spheroid formation after 4 days of culture (Fig. 4-a). When measured on day 7, the size of TSs (diameter) revealed a significant difference between mono-cultured TSs and TSs co-cultured with PSCs (71.9 vs. 67.5 μm, *p* < 0.05). Although the spheroids assumed a round shape under mono-culture conditions, co-cultured TSs had a significantly higher aspect ratio on days 6 and 7. Morphological changes in TSs manifested themselves in three distinct stages of progressive invasion of cancer cells (Fig. 4-b). The non-invasive stage was defined as all cells remaining within spheroids and having no membrane protrusion. Some TSs showing actin-rich membrane protrusions were defined as the invadopodium stage. Spheroids in the invasive stage showed cells moving out of spheroids and invading the surrounding matrix. After elongation of invadopodia, the nucleus of a leader cell relocated into invadopodia, streams of cells migrated out of spheroids, and eventually the spheroid structure disrupted. When the cells invaded the surrounding matrix, the nucleus appeared elongated compared to the cells remaining in the spheroids (Fig. 4-b). After 9 days of cultivation, 85% of TSs assumed the invasive stage under PSC co-culture conditions, showing 5-fold increase compared to 18% of invasive TSs under the mono-culture conditions (Fig. 4-c). Taken together, these data indicated that PSC co-culture accelerated invadopodia development and invasive migration of PANC-1 cells out of TSs.

### Induction of EMT in PANC-1 TSs by PSC co-culture

**Fig. 5:**
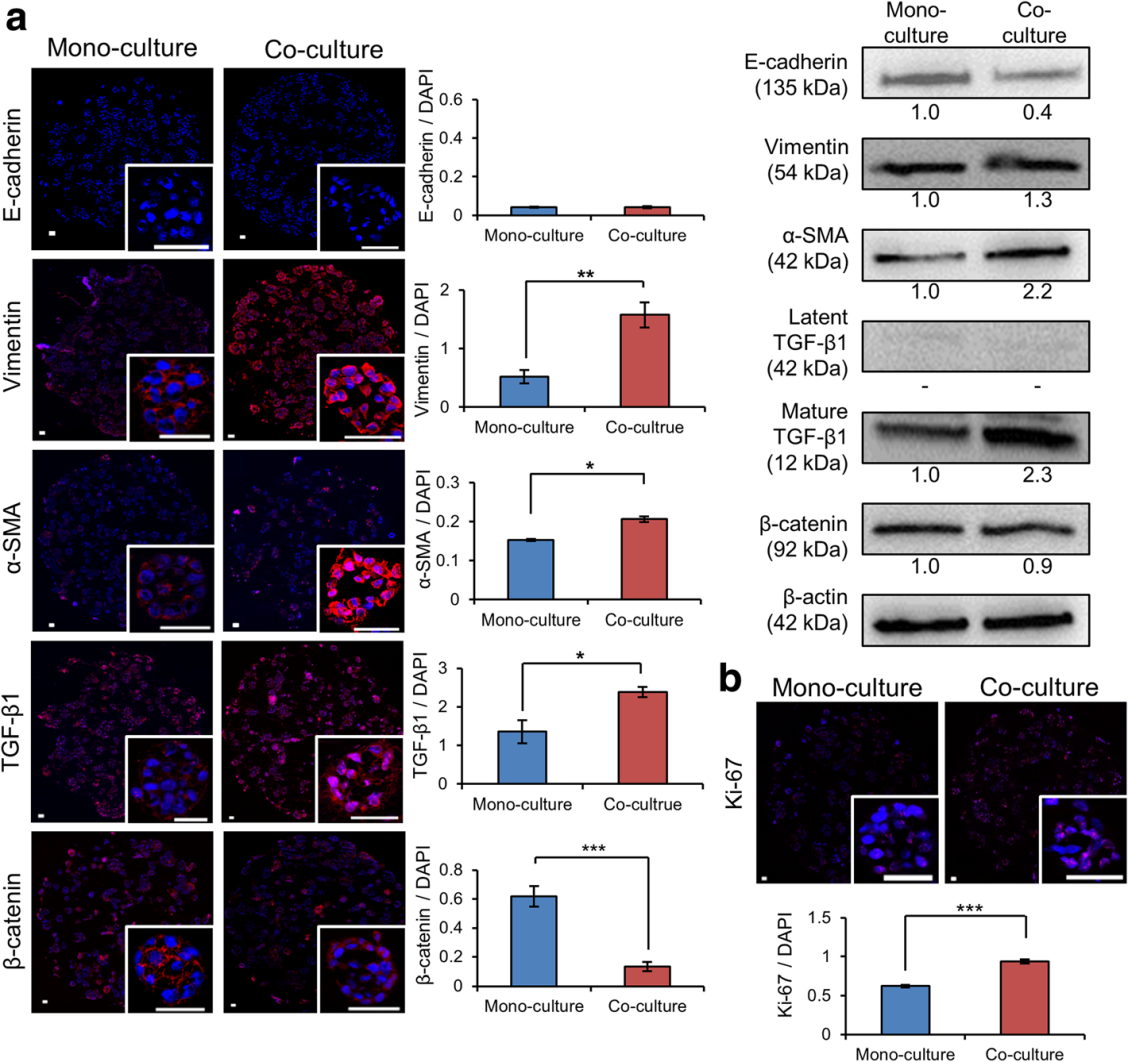
Increased expression of the EMT-related proteins in PANC-1 TSs under PSC co-culture conditions. **a** Changes in the expression levels of E-cadherin, vimentin, α-SMA, TGF-β1 and β-catenin as determined by immunofluorescence and western blot analysis. **b** Increased expression of Ki-67 under PSC co-culture conditions. Immunofluorescence staining was performed on cryo-sections of TSs following 7 days of culture. Optical sections were acquired at 1 μm intervals and stacked into a z-projection. Fluorescence intensity was measured from whole section of spheroid cultures and normalized by DAPI intensity. Individual TSs were shown in the insert. Data represent the mean ± SD of three independent experiments. Scale bars: 50 μm. * *p* < 0.05, **p < 0.01***p < 0.001

With ECM remodeling (Fig. 3) and increased invasion (Fig. 4) observed under PSC co-culture conditions, concurrent changes were seen in the expression levels of EMT-related factors (Fig. 5). Expression of mesenchymal markers such as vimentin and α-SMA increased along with the expression of TGF-β1, a major EMT inducer (Fig. 5-a). Although E-cadherin expression was too low to be detected by immunostaining of TSs, it was detectable by western blot analysis and decreased under PSC co-culture conditions. The decreased level of E-cadherin was accompanied by translocation of membrane-bound β-catenin to the cytosol as determined by immunostaining. Reduced immunofluorescence intensity of cytosolic β-catenin was observed, which may be attributed to incomplete fixation of soluble cytoplasmic proteins [23], a similar level of expression was seen in western blot analysis. Expression of a proliferation marker, Ki-67, significantly increased in PANC-1 TSs when they were co-cultured with PSCs (Fig. 5-b).

### Changes in the secretome under PSC co-culture conditions

**Fig. 6:**
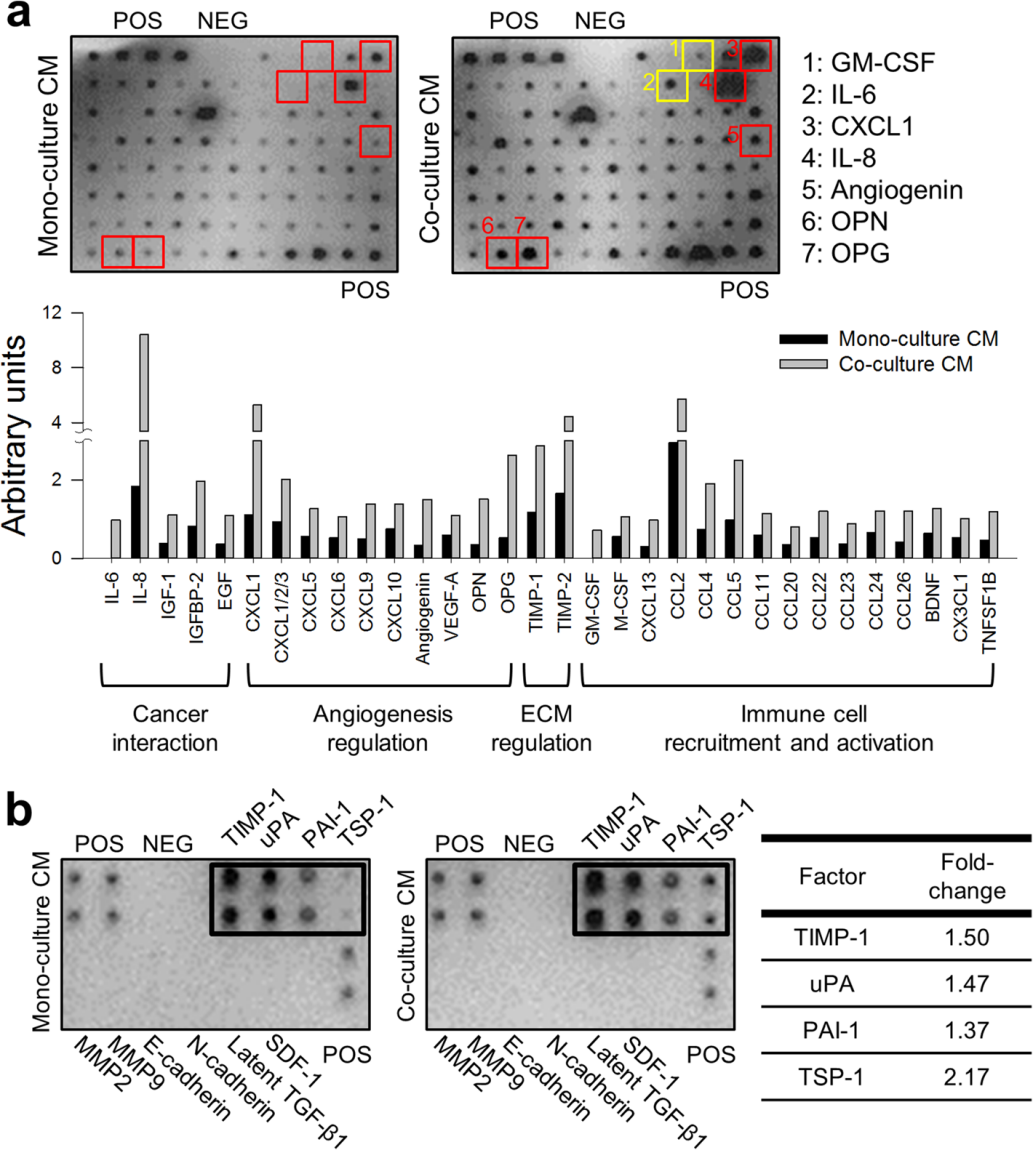
Increased secretion of chemokines and cytokines in CM of PANC-1 TS co-cultured with PSCs. **a** Representative images of human cytokine array analysis in CM of PANC-1 TS under mono-culture and PSC co-culture conditions. Two factors were detected only in co-culture CMs (yellow boxes); five showed over 5-fold increase under co-culture conditions (red boxes). In the graph are the factors showing increase by more than 2-folds. **b** Upregulation of the TGF-β1 and EMT-related cytokines in CM under TS-PSC co-culture conditions. CM: conditioned media; POS: positive control; NEG: negative control

From a secretome analysis, 32 out of 88 factors showed two- to five-fold increased level in the co-culture CM. Among these factors, five are known for their roles in the interaction with cancer cell such as interleukine-6 (IL-6) and IL-8, ten for angiogenesis regulation such as CXC-chemokine ligand-1 (CXCL-1), angiogenin and vascular endothelial growth factor-A (VEGF-A), two for matrix remodeling including TIMP-1 and TIMP-2, and fifteen for immune cell recruitment and activation such as macrophage colony-stimulating factor (M-CSF) and granulocyte M-CSF (GM-CSF) (Fig. 6-a). It was noted that IL-6 and GM-CSF were detected only in co-culture CM, suggesting exclusive secretion by PSCs. Hepatocyte growth factor (HGF), a well-known mediator of interaction between cancer cells and PSCs, showed no difference under PSC co-culture conditions.

In the analysis of the EMT-related factors in CM, four out of eight cytokines (except two cell adhesion molecules, E-cadherin and N-cadherin among 10 proteins) underwent significant upregulation under PSC co-culture conditions (Fig. 6-b). Four other factors including, MMP-2, MMP-9, Latent TGF-β1, and SDF-1 were not detectable in either condition. It is noted that TGF-β1 detected in PANC-1 TS was the mature isoform of 12 kDa fragment (Fig. 5-a), hence the latent form was not detectable by cytokine array assay (Fig. 6-b).

### Paclitaxel inhibits co-cultured cancer cell invasion by targeting PSC-mediated interactions

**Fig. 7:**
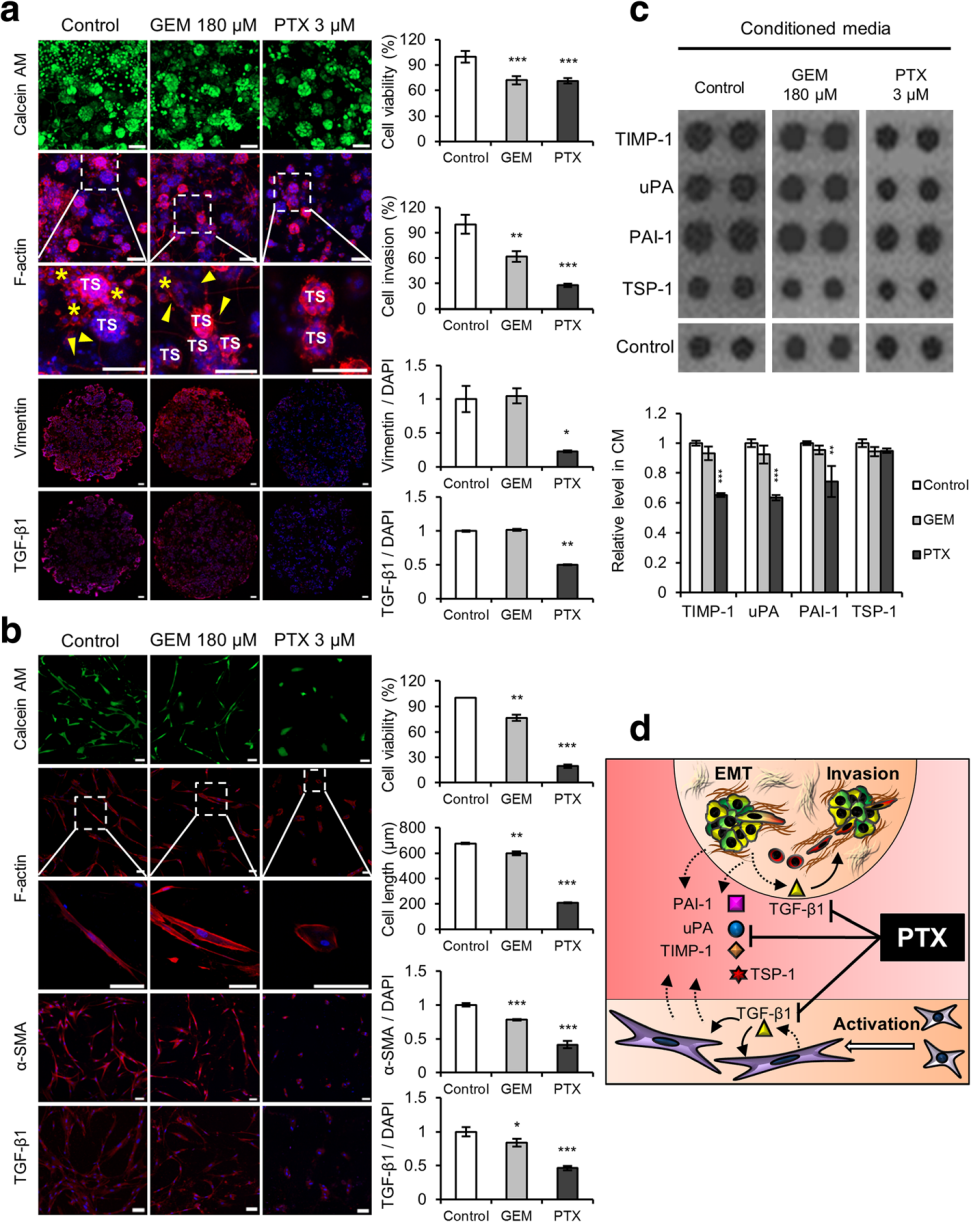
Paclitaxel inhibits the invasion of cancer cells and suppresses the viability of PSCs. **a** Changes in cell invasion and EMT factor expression in TSs when exposed to GEM or PTX. Staining was performed on whole TSs (calcein AM and F-actin) and cryo-sections (vimentin and TGF-β1). Yellow arrowhead and asterisk indicate invadopodia and dispersed single cells, respectively. **b** Changes in cell morphology and fibroblast activation factors of PSCs exposed to GEM or PTX. **c** Changes in the expression of four EMT-related cytokines in the CM following drug treatment. **d** Schematic illustration of the proposed mechanism of PTX-induced inhibition of reciprocal activation and cytokine cross-talk between TSs and PSCs. Optical sections were acquired at 1.5 μm intervals and stacked into a z-projection. Drug effect was compared at the concentrations of both drugs producing 30% decrease in viability (IC_30_) after 72 h exposure, i.e., 180 μM of GEM and 3 μM of PTX (Additional file 3: Figure S3-a). TS: tumor spheroid; GEM: gemcitabine; PTX: paclitaxel; CM: conditioned media. Data represent the mean ± SD of three independent experiments. Scale bars: 100 μm; *p < 0.05, **p < 0.01, ***p < 0.001 as compared to the control group

Dose–response curves of GEM and PTX were determined in groups of PANC-1 TSs grown in mono-culture or under PSC co-culture conditions (Additional file 3: Figure S3-a). It was noted that PSC co-culture raised resistance to PTX 100-fold in terms of IC_30_ (Mono-culture TSs IC_30_ = 0.033 μM; co-culture TSs IC_30_ = 3 μM), yet no difference was detected for GEM (Both mono- and co-culture TSs IC_30_ = 180 μM), indicating thet PANC-1 cells growns as spheroids were resistance to GEM. Anti-invasion activity was evaluated at a drug concentration producing 30% growth inhibition, i.e., 180 μM for GEM and 3 μM for PTX. Cell invasion was reduced by only 38% in the GEM-treated group, but by 72% in the PTX-treated group, indicating a superior anti-invasive activity of PTX, which was also supported by effective suppression of invadopodia formation in TSs (Fig. 7-a). Vimentin and TGF-β1 manifested significant downregulation in TSs after PTX exposure but not GEM, indicating that TGF-β1-mediated EMT signaling may be involved in the PTX-induced growth inhibition in (and anti-invasion effect on) PANC-1 TSs.

PSC exposure to GEM resulted in decreased viability (76% of the control) and cell length (88% of the control), which are similar to the magnitude of viability reduction (70% of control) in TSs (Fig. 7-b). On the other hand, PSCs being highly sensitive to PTX (3 μM) showed over 80 and 70% reductions in viability and cell length, respectively (Fig. 7-b and Additional file 3: Figure S3-b). In PSCs, reduced amounts of α-SMA and TGF-β1 were proportional to the drug-induced changes in viability and morphology for GEM and PTX treatment; these data point to the similar mechanisms of toxicity toward PSCs induced by these two drugs. Taken together, these data indicated that PTX, not GEM, exerted a strong inhibitory effect on cancer cell invasion and on proliferation of PSCs.

Among the four cytokines upregulated in the co-culture CM (Fig. 6-b), only three cytokines, namely TIMP-1, uPA, and PAI-1, after PTX exposure underwent significant downregulation approaching the level determined in the absence of PSCs (Fig. 7-c). Despite the inhibitory effect of GEM on PSC viability, no significant change was observed in the amounts of the four cytokines. Collectively, these data indicated that PTX exerted a superior effect on cancer cell invasion as compared to GEM. In the underlying mechanism, TIMP-1, uPA, and PAI-1, but not TSP-1, may be involved (Fig. 7-d).

## Discussion

In this study, we established an in vitro tumor model that recapitulates the TME of PDAC. Based on our previously developed method of hydrogel-embedded 3D cell culture by means of a minipillar array chip [18, 19], the culture method was modified to facilitate simultaneous reciprocal interactions between cancer cells and PSCs. We utilized a separate (indirect) co-culture method in which respective cell populations can be monitored without using either a particular separation procedure or specific cell probes [24]. Changes in the phenotypic and molecular signatures of TSs and PSCs were evaluated either by in-well staining followed by optical sectioning microscopy or by conventional slide staining of mechanical sections (cryo-sections and paraffin-embedded sections), for which our pitch-tunable minipillar array chip provides a most suitable platform [18, 19]. Mechanical sections are useful when either limited penetration of antibodies or light scattering hinders proper staining or 3D imaging, respectively, within a dense 3D architecture of a TS. By optimizing the culture conditions, sample preparation, and the 3D imaging protocol, we were able to analyze ECM remodeling, cell invasion, and drug resistance induced by 3D interactions between cancer cells and PSCs in an efficient and clinically relevant way.

Several pancreatic cancer cell lines are commercially available and have been characterized regarding their different mesenchymal states [25, 26]. We confirmed the differential profiles of E-cadherin and vimentin expression among five PDAC cell lines, i.e., MIAPaCa-2, AsPC-1, PANC-1, Capan-1, and BxPC-3 (Fig. 1-b). A correlation between formation or compactness of TSs and the level of E-cadherin expression has been observed for these cell lines. When growing 3D in ultra-low attachment plates, Capan-1 and BxPC-3 cells were aggregated to form spheroids, whereas MIAPaCa-2, AsPC-1, and PANC-1 cells did not (Additional file 4: Figure S4). When cultured in a collagen gel using minipillar chips, each of the five cell lines aggregated to form TSs, yet the surface roughness appeared to reflect their EMT states (Fig. 1-b). A similar relation was demonstrated for head and neck cancer cells grown by liquid overlay methods and for breast cancer cells grown embedded in a hydrogel [27, 28]. In addition, drug sensitivity has been linked to the EMT status of cells [25]. Mesenchymal-type PDAC cells showed higher resistance to GEM as compared to the epithelial type in monolayers (MIAPaCa-2, AsPC-1, and PANC-1 cells: IC_50_ ≥ 10 μM; Capan-1 and BxPC-3 cells: IC_50_ ≤ 0.1 μM; Additional file 5: Figure S5) as well as in 3D cultures (PANC-1 TSs, IC_30_ = 180 μM; BxPC-3 TSs, IC_30_ ≤ 10 μM; Additional files 3 and 6: Figure S3-a, S6).

PSCs were activated when co-cultured with TS in our 3D model (Fig. 2). Expression level of vimentin and Wnt2 was significant, but was not affected by interaction with TS (Additional file 7: Figure S7), whereas morphologic changes to a further elongated spindle shape with stress fiber formation was observed with increased expression of α-SMA and TGF-β1 under TS co-culture conditions (Fig. 2). Activated PSCs act as CAFs to promote cancer cell proliferation and migration [29]. CAFs induce matrix deposition and proteolytic remodeling of ECM by producing proteases and their inhibitors such as TIMP-1, TIMP-2 and uPA (Fig. 6) [30]. CAFs also stimulate cancer cell migration via paracrine signaling of IL-6, IL-8, insulin like growth factor-1 (IGF-1), CCL2, CCL4, CCL5, CCL22, CXCL1, CXCL2, CXCL3, CXCL5, CXCL6, TSP-1, PAI-1 osteopontin (OPN) and osteoprotegerin (OPG), all of which were shown to increase in the CM under PSC co-culture conditions (Fig. 6) [31, 32].

Due to tumor-promoting effect, targeting CAFs with Hedgehog (Hh) inhibitors has been considered a promising anti-tumor strategy [33]. Clinical trials evaluating Hh inhibitors, however, failed to show clinical benefit in PDAC whereas two Hh inhibitors (inhibiting smoothened) [34], vismodegib and sonidegib have been approved to treat basal cell carcinomas [35]. Although the role of Hh signaling pathway in PDAC and CAFs’ functional heterogeneity remains controversial [36, 37], therapeutic potential of stromal CAF targeting warrants further studies using more clinically relevant models [38].

Cancer cell invasion was accompanied by ECM remodeling when TSs were co-cultured with PSCs (Figs. 3 and 4). In TSs co-cultured with PSCs, formation of fibrous ECM bundles may be attributed to changes in collagen fiber orientation (Fig. 3); this notion supports the usefulness of our model for studying the plasticity of matrix and reciprocal of cell-matrix interactions [39]. The mechanism underlying this ECM remodeling involves matrix degradation by proteases such as MMPs (Additional file 2: Figure S2-b) and ADAMs (A disintegrin and metalloproteinases) secreted by cancer cells and PSCs and internalization by collagen receptor uPARAP/Endo180 followed by deposition of the fibrillar form of collagen [40, 41]. Increased deposition of fibrous matrix proteins as part of ECM remodeling has been linked to reduced drug accumulation in tumor tissue [42, 43]. In our co-culture model, doxorubicin penetration was reduced in PANC-1 TSs by co-culturing with PSCs (Additional file 8: Figure S8), indicating that excessive ECM deposition acted as a physical barrier to diffusion or convective movement of drug molecule. Under PSC co-culture conditions, cancer cell invasion measured by dispersed single cell numbers was accompanied by increase in aspect ratio of TS (Fig. 4). The changes in the aspect ratio were not due to increase in size or cell death in either mono- and co-culture conditions (Additional file 9: Figure S9).

One of the mechanisms via which TGF-β1 enhances mesenchymal traits in cells is to produce several secreted proteins including growth factors [44]. TIMP-1, uPA, and PAI-1 are some of the mediators of TGF-β1-induced EMT in cancer [45, 46]. TIMP-1 is associated, independently of its MMP-inhibitory function, with the induction of the EMT phenotype and mediates cancer-stellate cell interactions [47]. uPA plays an important role in ECM remodeling by converting plasminogen to plasmin for direct degradation of ECM components or activates latent TGF-β1 [48]. PAI-1 is known as the main inhibitor of the uPA-uPA receptor (uPAR) complex but is overexpressed in CAFs and enhances cell migration in an uPA-uPAR-independent manner [49, 50]. TSP-1, a major inducer of TGF-β1 [51], is expressed in CAFs surrounding tumor cells and is involved in the regulation of ECM remodeling during tumor invasion [52, 53]. Changes in the EMT signature of cancer cells and PSCs were accompanied by increased expression of TGF-β1 (Figs. 2-b, 5-a). We also observed increased concentrations of four TGF-β1-related soluble factors such as TSP-1, uPA, PAI-1, and TIMP-1 in the CM under PSC co-culture conditions (Fig. 6-b). Thus, it is demonstrated that our model recapitulates the ‘reciprocal interactions’ between cancer cells and stellate cells, through which mutual activation was induced and resulted in the secretion of cytokines for enhancement of ECM remodeling and cell invasion.PTX caused significant suppression of cancer cell invasion as compared to GEM in PANC-1 TSs co-cultured with PSCs (Fig. 7-a). PTX is known to bind to and stabilize microtubules thereby inhibiting their dynamics and resulting in mitotic arrest and cell death [54]. Nonetheless, the anti-invasive effect of PTX observed in the present study can be explained by the mitosis-independent mechanism in both PANC-1 cancer cells and PSCs. Microtubules are known to cooperate with actin and vimentin intermediate filaments for elongation of invadopodia [21]. Hence, microtubule-stabilizing effect of PTX significantly suppresses cytoskeleton network remodeling in invadopodia maturation and subsequent cancer cell invasion (Fig. 7-a). In addition, the microtubule network negatively regulates TGF-β signaling by sequestering Smads [55, 56]. Hence, reduced mesenchymal signature in PANC-1 TSs and PSCs after PTX exposure (Fig. 7) can be explained by inhibition of TGF-β-Smad signaling via PTX-induced microtubule stabilization. PTX-induced inhibition of TGF-β signaling was reflected in the changes of secreted cytokine levels after PTX exposure, where mediators of TGF-β signaling such as TIMP-1, uPA, and PAI-1 underwent significant downregulation, but not TSP-1, a major activator of TGF-β1 [51], (Figs. 7-c, d). From the standpoint of non-mitotic anti-invasion activity of PTX, the mechanism behind the clinical synergism between albumin-bound PTX (nab-PTX) and GEM is worth further investigation [57].

## Conclusion

We established co-culture of pancreatic TSs and PSCs by means of minipillar array chips and present it here as a novel in vitro model of a human PDAC. Our model recapitulates the 3D interactions between cancer-cancer cells, cancer cell-ECM interactions, and cancer cell-PSC interactions. Our model is demonstrated to be useful for studying the effects of PSCs on ECM remodeling and cancer cell invasion and the mechanisms involved. Drug response was successfully analyzed in a cell type-specific and quantitative manner. Overall, our co-culture model may represent a novel method not only for studying TME interactions but also for evaluating therapeutic agents targeting these interactions [58–61].

## Additional files

Additional file 1: **Figure S1.** Components of the minipillar array chip. The minipillar array chip consists of a pair of base chips (top and bottom chip) (a) and minipillars (b). (c) Assembly of pillars are shown as positioned at 9 mm distance or 3.2 mm distance. (US Patent Application No. 15/347,767, ROK Patent Registration No. 10–1,860,502) (TIF 1236 kb)

Additional file 2: **Figure S2.** Expression of vimentin (a) and MT1-MMP (b) in invadopodia of PANC-1 TSs. In-well staining was carried out for vimentin and MT1-MMP (green), F-actin (red) and DAPI (blue) in whole TSs. Optical sections were acquired at 0.5 μm intervals and stacked into a z-projection. Scale bars: 20 μm. (TIF 1254 kb)

Additional file 3: **Figure S3**. Differential sensitivity of PANC-1 TSs and PSCs to anticancer drugs. Dose-response curves of GEM and PTX for PANC-1 TSs (a) and PSCs (b) was determined under mono- or co-culture conditions after 72 h exposure by APH assay. Data represent the mean ± SD of three independent experiments. (TIF 146 kb)

Additional file 4: **Figure S4.** The spheroid formation of pancreatic cancer cells when cultured in ultra-low attachment plates. Cells were seeded at 3 × 10^3^ cells/well in 96-well ultra-low attachment plates. Cellular aggregation and morphology was monitored under bright field microscopy over 6 days of culture. Scale bars: 500 μm. (TIF 1128 kb)

Additional file 5: **Figure S5**. Differential sensitivity to GEM in pancreatic cancer cell lines when cultured as monolayers in 96-well plates. Drug-response was measured after 72 h exposure using APH assay. Data represent the mean ± SD of three independent experiments. (TIF 50 kb)

Additional file 6: **Figure S6.** Effect of PSC co-culture on GEM sensitivity of BxPC-3 cells grown as TSs. Dose-response curves of GEM was determined under mono- or co-culture conditions after 72 h exposure by APH assay. Data represent the mean ± SD of three independent experiments. (TIF 39 kb)

Additional file 7: **Figure S7.** Expression of vimentin and Wnt2 in PSCs under mono- or co-culture with PANC-1 TSs. Immunostaining was done after 7 day of culture in 96-well plates. Optical sections were acquired at 1.5 μm intervals and stacked into a z-projection. Data represent the mean ± SD of three independent experiments. Scale bars: 200 μm. (TIF 779 kb)

Additional file 8: **Figure S8.** Comparison of doxorubicin accumulation in mono- or co-cultured PANC-1 TSs. A drug uptake was measured after 1 h exposure at indicated concentrations. Optical sections were acquired at 1 μm intervals and stacked into a z-projection on pillar tips. Data represent the mean ± SD of three independent experiments. Scale bars: 50 μm. (TIF 421 kb)

Additional file 9: **Figure S9.** Changes in spheroid aspect ratio by PSC co-culture (Fig. 4-a) was not due to spheroid size or cell death. (a) Aspect ratios of PANC-1 TSs showed no relationship with spheroid size in both mono- and co-culture conditions. (b) No difference in cell viability of PANC-1 TSs under mono- or co-culture of PSCs. PANC-1 TSs were grown in the absence and presence of PSCs for 7 days. Staining of whole TSs was carried out during cultivation in the well plates, and optical sections were acquired at 10 μm intervals and stacked into a z-projection. Data represent the mean ± SD of three independent experiments. Scale bars: 200 μm. (TIF 1375 kb)

## Abbreviations

2D: Two-dimensional
3D: Three-dimensional
BDNF: Brain-derived neutrophic factor
CAFs: Cancer-associated fibroblasts
CCL: CC-chemokines ligand
CM: Conditioned media
CX_3_CL: CX_3_C-chemokines ligand
CXCL: CXC-chemokines ligand
E-cadherin: Epithelial-cadherin
EGF: Epithelial growth factor
EMT: Epithelial-mesenchymal transition
GEM: Gemcitabine
GM-CSF: Granulocyte macrophage colony-stimulating factor
HGF: Hepatocyte growth factor
Hh: Hedgehog
IGF-1: Insulin like growth factor-1
IGFBP-2: Insulin like growth factor binding protein-2
IL-6: Interleukin-6
IL-8: Interleukin-8
M-CSF: Macrophage colony-stimulating factor
MMP-2: Matrix metalloproteinase-2
MMP-9: Matrix metalloproteinase-9
MT1-MMP: Membrane-type 1 matrix metalloproteinase
N-cadherin: Neural-cadherin
OPG: Osteoprotegerin
OPN: Osteopontin
PAI-1: Plasminogen activator inhibitor-1
PDAC: Pancreatic ductal adenocarcinoma
PSCs: Pancreatic stellate cells
PTX: Paclitaxel
SDF-1: Stromal cell derived factor-1
TGF-β1: Transforming growth factor beta-1
TIMP: Tissue inhibitors of metalloproteinases
TME: Tumor microenvironment
TNFSF1B: Tumor-necrosis factor super family-1B
TSP-1: Thrombospondon-1
TSs: Tumor spheroids
uPA: Urokinase plasminogen activator
uPAR: Urokinase plasminogen activator receptor
VEGF-A: Vascular endothelial growth factor-A
Wnt2: Wingless-related integration site-2
α-SMA: α-Smooth muscle actin
