# Supplementary figures and images for: Multiplex quantitative analysis of stroma-mediated cancer cell invasion, matrix remodeling, and drug response in a 3D co-culture model of pancreatic tumor spheroids and stellate cells
Source: J Exp Clin Cancer Res. 2019 Jun 14;38:258. doi: 10.1186/s13046-019-1225-9 (PMC6567511; doi:10.1186/s13046-019-1225-9)

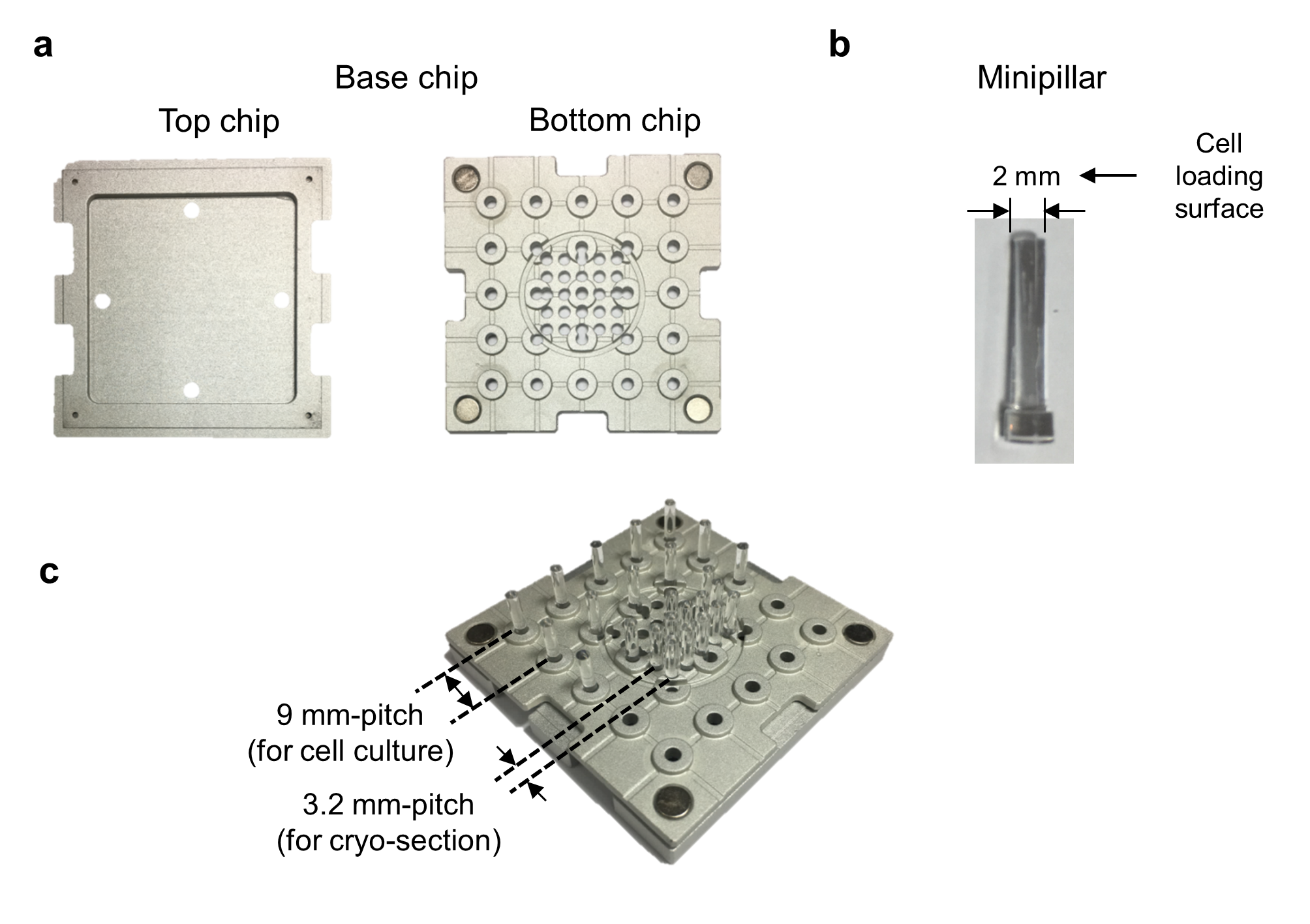

Supplement: Supplementary file 1 — Figure S1. Components of the minipillar array chip. The minipillar array chip consists of a pair of base chips (top and bottom chip) (a) and minipillars (b). (c) Assembly of pillars are shown as positioned at 9 mm distance or 3.2 mm distance. (US Patent Application No. 15/347,767, ROK Patent Registration No. 10–1,860,502) (TIF 1236 kb) [file 13046_2019_1225_MOESM1_ESM.tif]

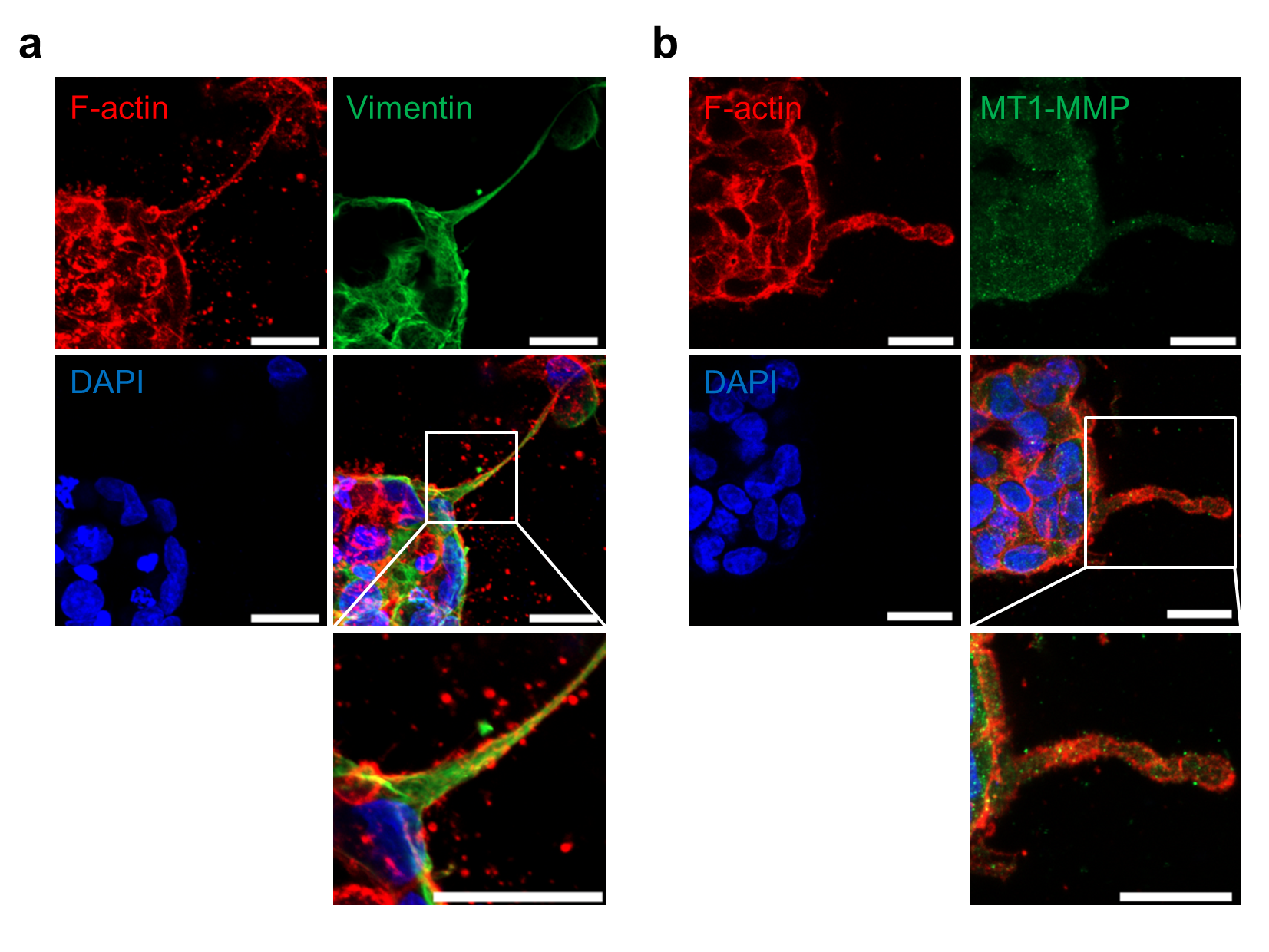

Supplement: Supplementary file 2 — Figure S2. Expression of vimentin (a) and MT1-MMP (b) in invadopodia of PANC-1 TSs. In-well staining was carried out for vimentin and MT1-MMP (green), F-actin (red) and DAPI (blue) in whole TSs. Optical sections were acquired at 0.5 μm intervals and stacked into a z-projection. Scale bars: 20 μm. (TIF 1254 kb) [file 13046_2019_1225_MOESM2_ESM.tif]

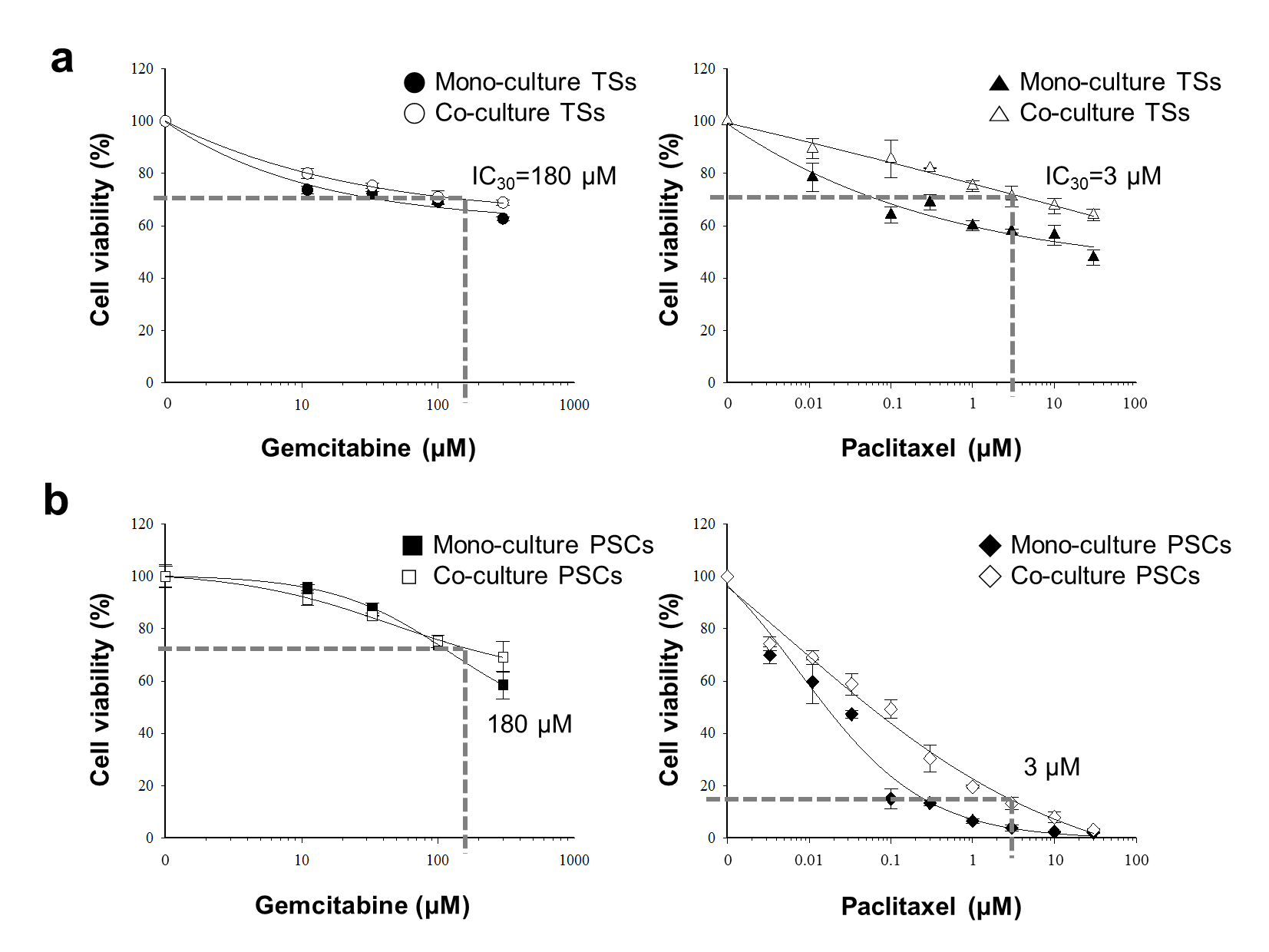

Supplement: Supplementary file 3 — Figure S3. Differential sensitivity of PANC-1 TSs and PSCs to anticancer drugs. Dose-response curves of GEM and PTX for PANC-1 TSs (a) and PSCs (b) was determined under mono- or co-culture conditions after 72 h exposure by APH assay. Data represent the mean ± SD of three independent experiments. (TIF 146 kb) [file 13046_2019_1225_MOESM3_ESM.tif]

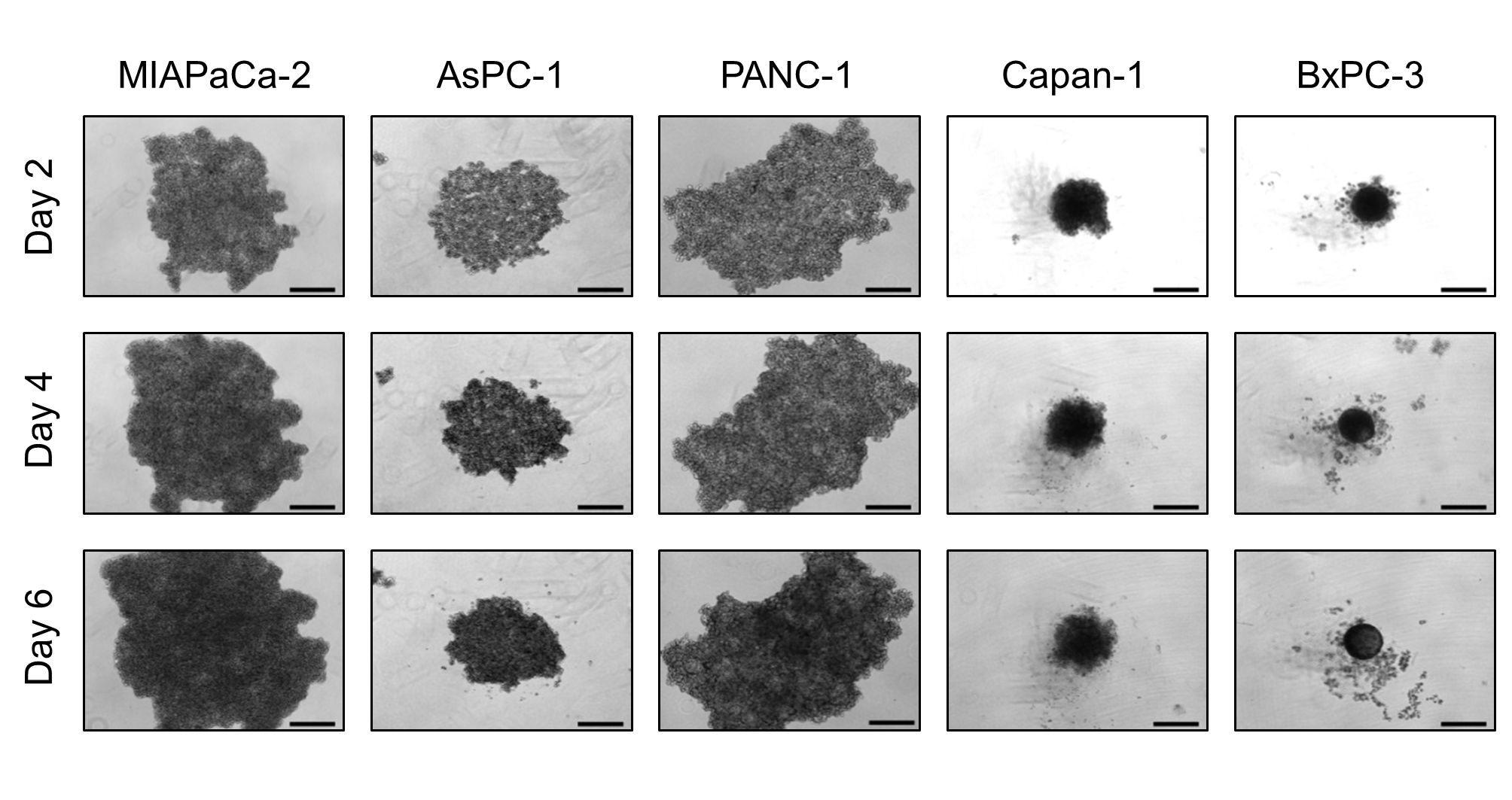

Supplement: Supplementary file 4 — Figure S4. The spheroid formation of pancreatic cancer cells when cultured in ultra-low attachment plates. Cells were seeded at 3 × 10^3^ cells/well in 96-well ultra-low attachment plates. Cellular aggregation and morphology was monitored under bright field microscopy over 6 days of culture. Scale bars: 500 μm. (TIF 1128 kb) [file 13046_2019_1225_MOESM4_ESM.tif]

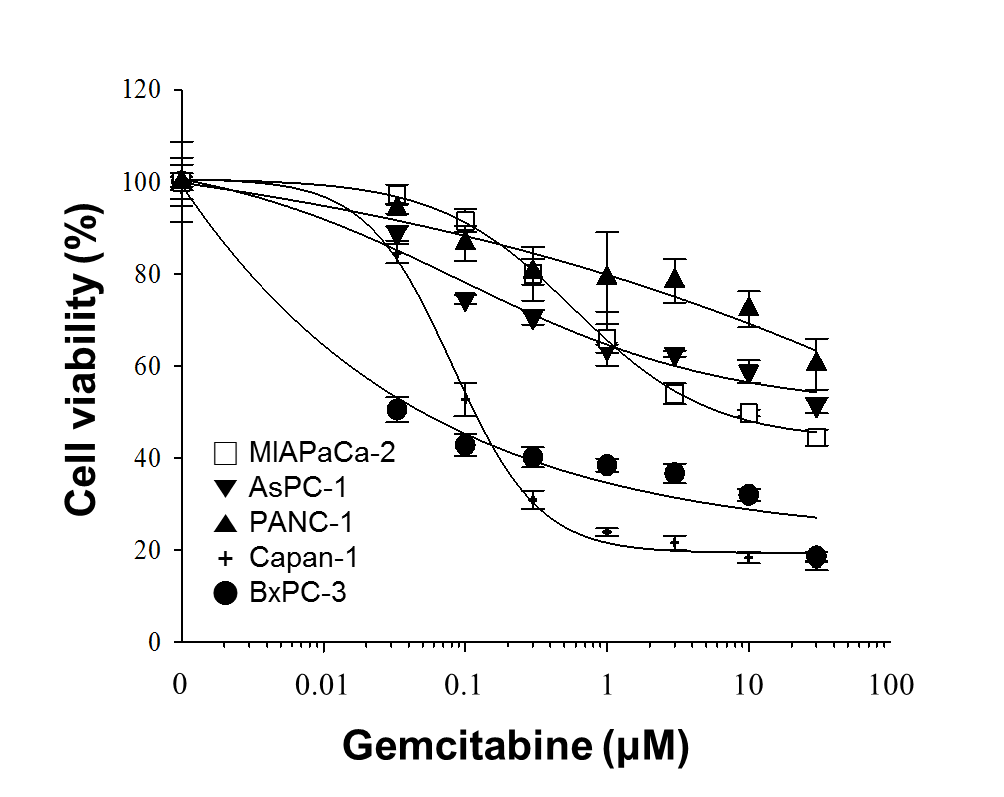

Supplement: Supplementary file 5 — Figure S5. Differential sensitivity to GEM in pancreatic cancer cell lines when cultured as monolayers in 96-well plates. Drug-response was measured after 72 h exposure using APH assay. Data represent the mean ± SD of three independent experiments. (TIF 50 kb) [file 13046_2019_1225_MOESM5_ESM.tif]

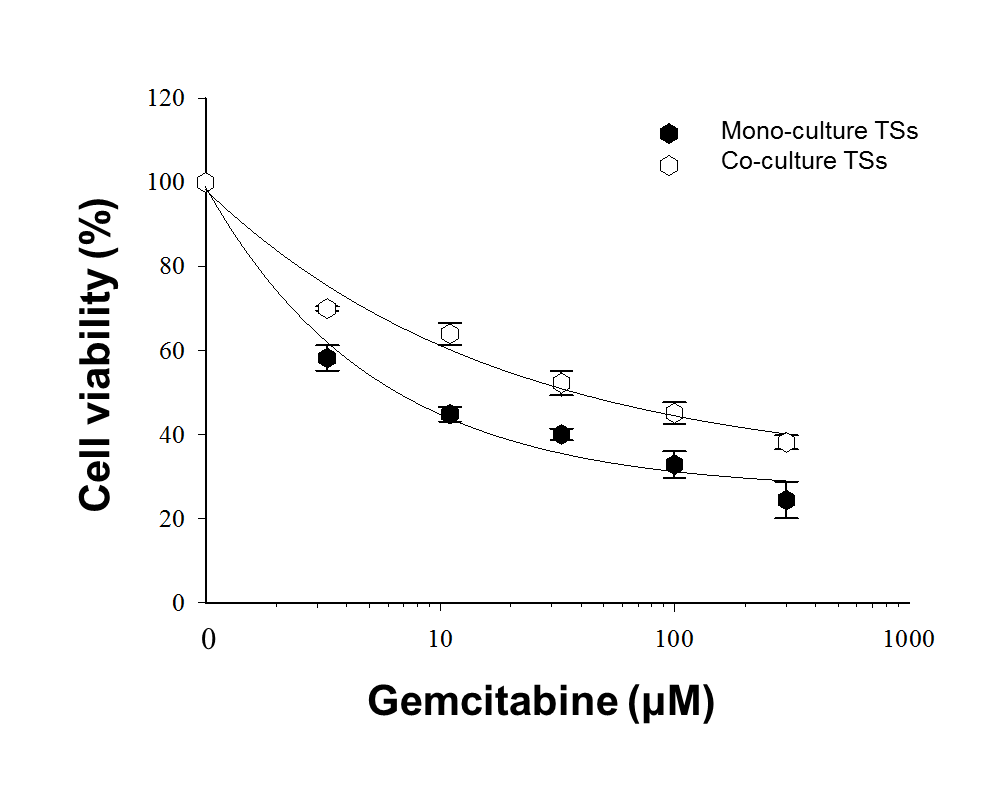

Supplement: Supplementary file 6 — Figure S6. Effect of PSC co-culture on GEM sensitivity of BxPC-3 cells grown as TSs. Dose-response curves of GEM was determined under mono- or co-culture conditions after 72 h exposure by APH assay. Data represent the mean ± SD of three independent experiments. (TIF 39 kb) [file 13046_2019_1225_MOESM6_ESM.tif]

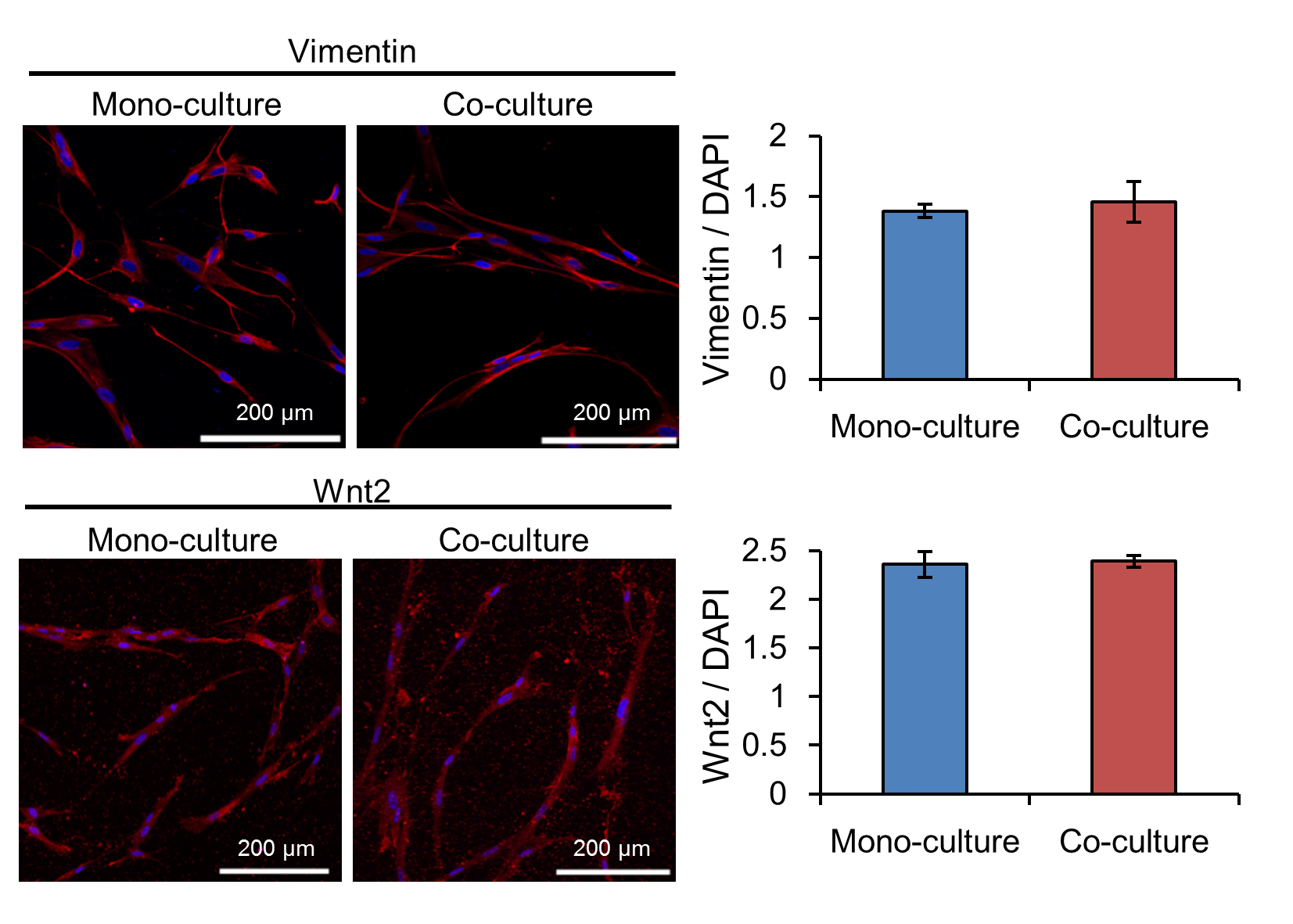

Supplement: Supplementary file 7 — Figure S7. Expression of vimentin and Wnt2 in PSCs under mono- or co-culture with PANC-1 TSs. Immunostaining was done after 7 day of culture in 96-well plates. Optical sections were acquired at 1.5 μm intervals and stacked into a z-projection. Data represent the mean ± SD of three independent experiments. Scale bars: 200 μm. (TIF 779 kb) [file 13046_2019_1225_MOESM7_ESM.tif]

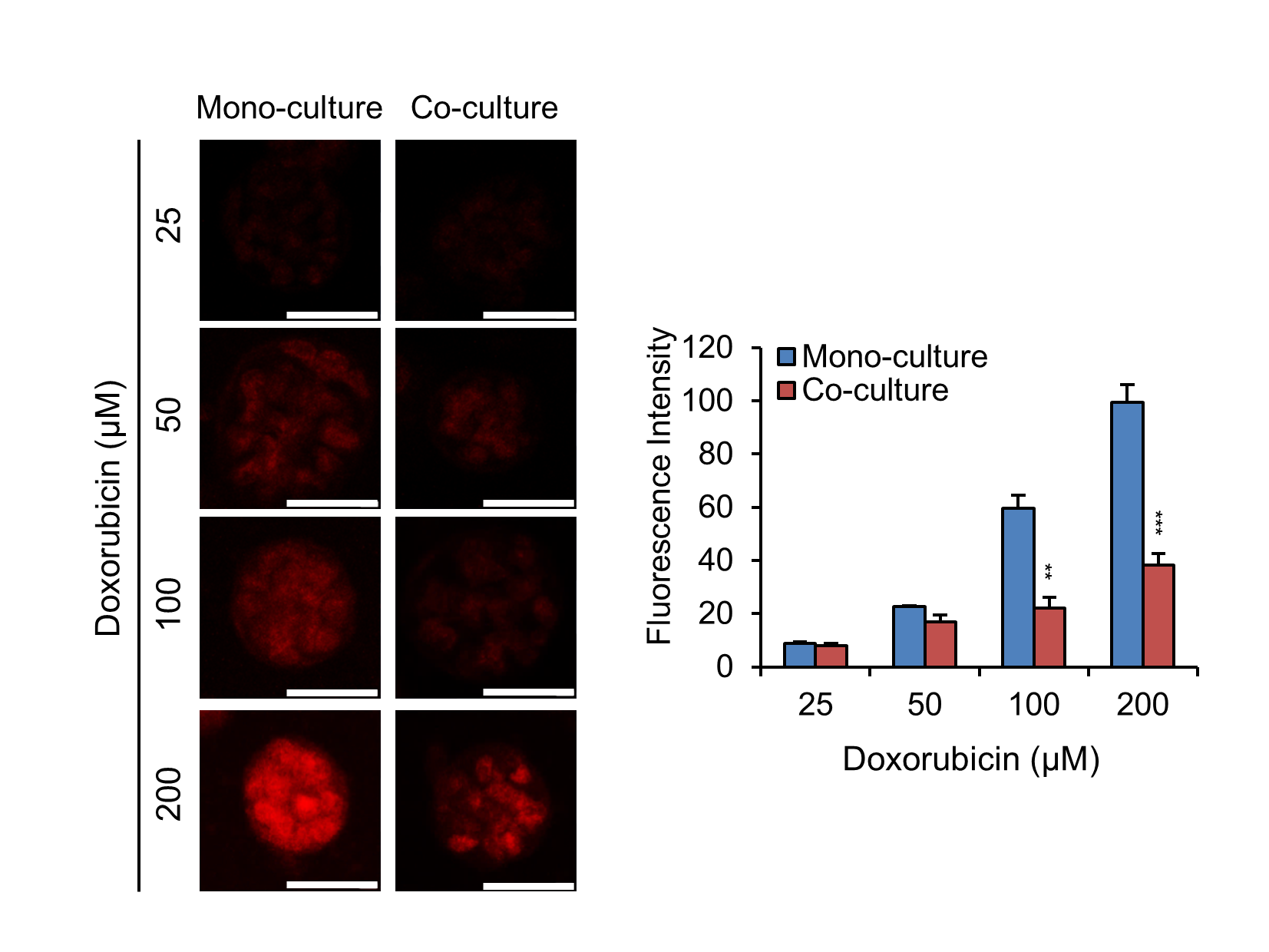

Supplement: Supplementary file 8 — Figure S8. Comparison of doxorubicin accumulation in mono- or co-cultured PANC-1 TSs. A drug uptake was measured after 1 h exposure at indicated concentrations. Optical sections were acquired at 1 μm intervals and stacked into a z-projection on pillar tips. Data represent the mean ± SD of three independent experiments. Scale bars: 50 μm. (TIF 421 kb) [file 13046_2019_1225_MOESM8_ESM.tif]

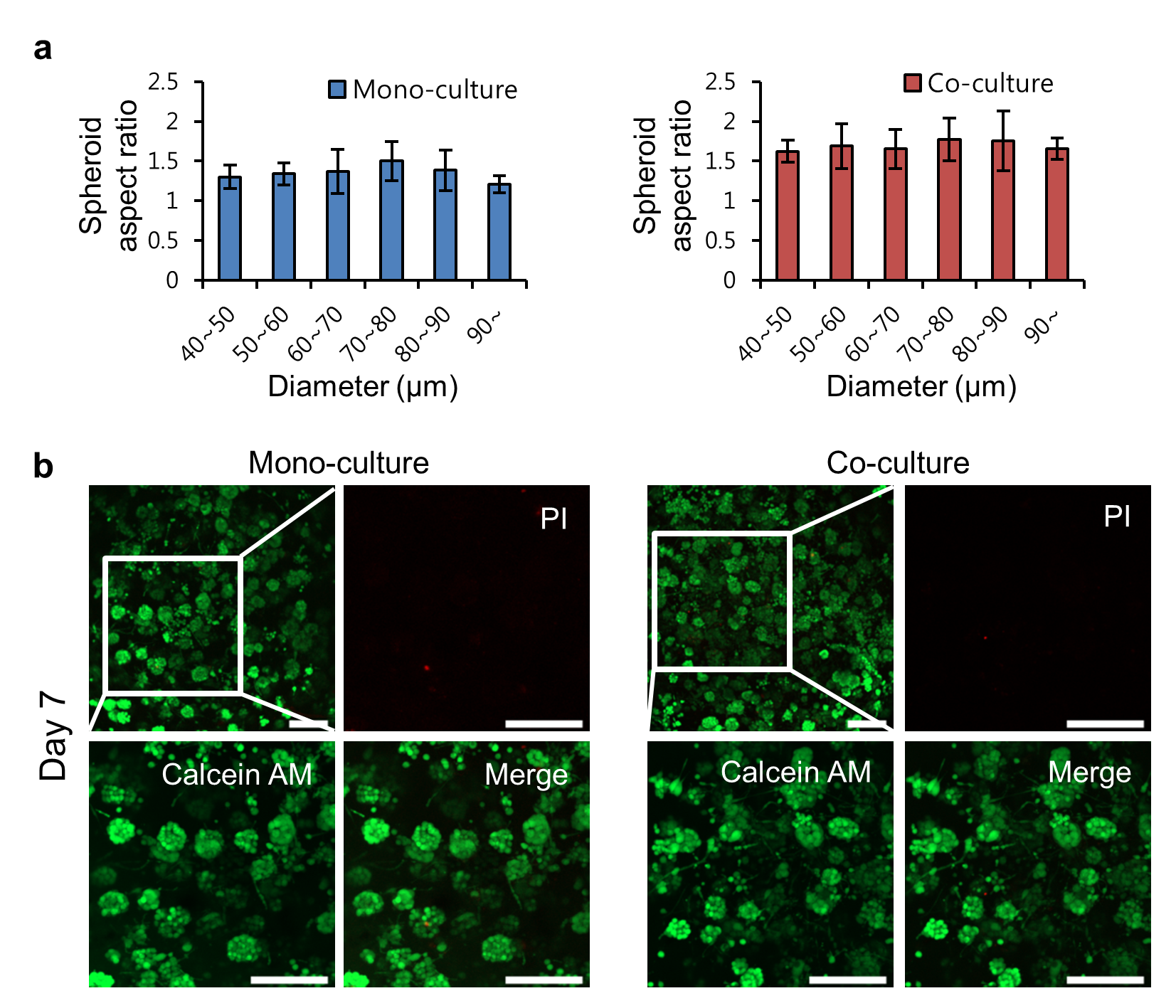

Supplement: Supplementary file 9 — Figure S9. Changes in spheroid aspect ratio by PSC co-culture (Fig. 4-a) was not due to spheroid size or cell death. (a) Aspect ratios of PANC-1 TSs showed no relationship with spheroid size in both mono- and co-culture conditions. (b) No difference in cell viability of PANC-1 TSs under mono- or co-culture of PSCs. PANC-1 TSs were grown in the absence and presence of PSCs for 7 days. Staining of whole TSs was carried out during cultivation in the well plates, and optical sections were acquired at 10 μm intervals and stacked into a z-projection. Data represent the mean ± SD of three independent experiments. Scale bars: 200 μm. (TIF 1375 kb) [file 13046_2019_1225_MOESM9_ESM.tif]
